# Development and Application of a Species–Specific eDNA–Based qPCR Assay for Early Detection of the Invasive Ascidian *Ascidiella aspersa*

**DOI:** 10.1002/ece3.72453

**Published:** 2025-11-10

**Authors:** Jeounghee Lee, Seongjae Kim, Soyeon Kwon, Jinho Lee, Sook Shin

**Affiliations:** ^1^ Marine Animal Biodiversity Center Sahmyook University Seoul Republic of Korea; ^2^ Division of EcoScience Ewha Womans University Seoul Republic of Korea; ^3^ Department of Animal Resources Science Sahmyook University Seoul Republic of Korea

**Keywords:** *Ascidiella aspersa*, early detection, environmental DNA, harbor monitoring, invasive species, Korea, qPCR assay

## Abstract

The solitary ascidian *Ascidiella aspersa* is an emerging invasive species in coastal ecosystems worldwide, including Korean waters. We developed and validated a species‐specific quantitative PCR (qPCR) assay targeting the mitochondrial COI gene, showing high specificity against 128 non‐target taxa and robust performance (Efficiency = 110.1%, *R* ^2^ = 0.9962, LOD = 12 copies/reaction, LOQ = 598 copies/reaction). The assay was applied to over 300 environmental DNA (eDNA) samples collected across 18 harbors between 2019 and 2021, and to an additional survey in 2022 that targeted four representative harbors (Bieung, Tongyeong, Yangpo, and Jeju) selected from the East, West, South, and Jeju Seas. Seasonal detection patterns showed peak DNA concentrations during summer, coinciding with the reproductive season of *A. aspersa* . Geographic analysis revealed a broader distribution than previously recorded, including new detections in Jeju Island and eastern ports. These findings highlight the assay's utility for early detection, risk assessment, and surveillance of *A. aspersa* , supporting its integration into national biosecurity frameworks. This study demonstrates the power of eDNA‐based diagnostics for managing marine invasive species through scalable and non‐invasive monitoring strategies.

## 1 Introduction

Marine ecosystems face increasing pressure from climate change, habitat degradation, and the spread of non–indigenous species (NIS), resulting in biodiversity loss, altered ecosystem functioning, and economic damage (Ojaveer et al. 2015; Seebens et al. 2018; López‐Gappa 2022). Among benthic invertebrates, ascidians are often dominant components of fouling communities and play important roles in ecological succession (Lindeyer and Gittenberger 2011; Lambert 2001; Soares et al. 2022; Van Deurs et al. 2021). However, several ascidian species have expanded far beyond their native ranges through human–mediated vectors such as shipping and aquaculture, leading to significant ecological impacts (Kaplan et al. 2018; Lins et al. 2018; Rodríguez et al. 2025; Zenetos et al. 2022).

**FIGURE 1:**
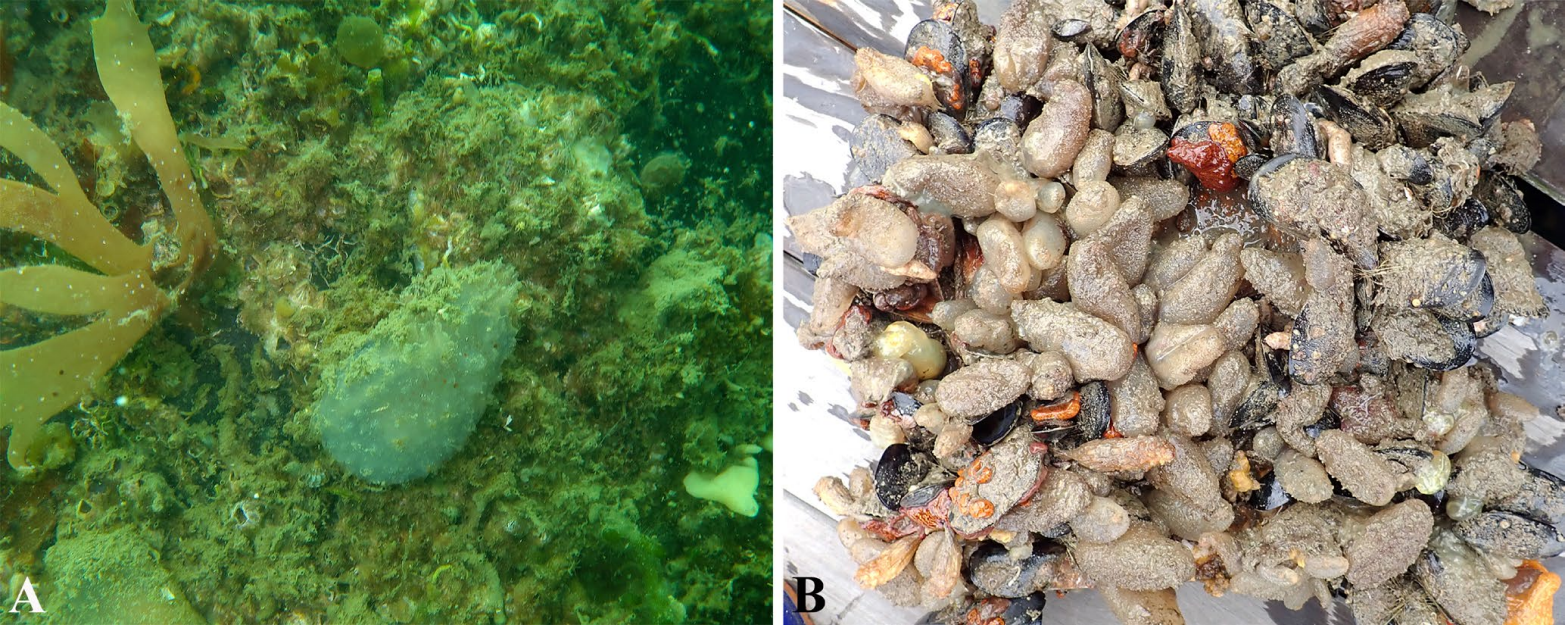
Morphological characteristics of *Ascidiella aspersa* . (A) *A. aspersa* specimens attached to harbor infrastructure. (B) Colonies of *A. aspersa* documented during settlement plate monitoring surveys.

The solitary ascidian *Ascidiella aspersa* is one of the most successful invasive species, exhibiting high reproductive capacity and tolerance to a wide range of environmental conditions (Figure 1) (Lynch et al. 2016; Pyo et al. 2012; Lee et al. 2023). Its global spread has been documented across temperate coastal regions, often outcompeting native taxa and altering fouling community composition. These patterns highlight the need for early detection and rapid response to reduce ecological risk and prevent the establishment of new populations (Hoffman 2020; Lindeyer and Gittenberger 2011; Rodríguez et al. 2025).

Traditional monitoring methods such as trawling, SCUBA surveys, and morphological identification are often insufficient for detecting low–abundance or cryptic species (Danovaro et al. 2016; Jørgensen et al. 2011; Pohle and Thomas 2001). In contrast, environmental DNA (eDNA) analysis enables species detection based on trace genetic material in water samples, providing high sensitivity without physical specimen collection (Doyle and Uthicke 2021; LeBlanc et al. 2020; Rodriguez‐Ezpeleta et al. 2021). eDNA metabarcoding and quantitative PCR (qPCR) assays have improved biodiversity assessment and invasive species monitoring, enabling integration with ecosystem surveys to reveal previously overlooked distributions (Closek et al. 2025; Pastor Rollan et al. 2024; Thomsen and Willerslev 2015; Wang et al. 2023).

In this study, we developed a patented species‐specific qPCR assay targeting *A. aspersa* (KR 10–2023–0000688) and applied it to a nationwide harbor monitoring program. This study develops and validates a species‐specific eDNA qPCR assay for *A. aspersa* and demonstrates its field application across multiple Korean coastal sites. Our approach demonstrates how species‐specific molecular tools can strengthen national biosecurity frameworks and inform management strategies.

## 2 Methods

### 2.1 Study Sites and Sampling

**FIGURE 2:**
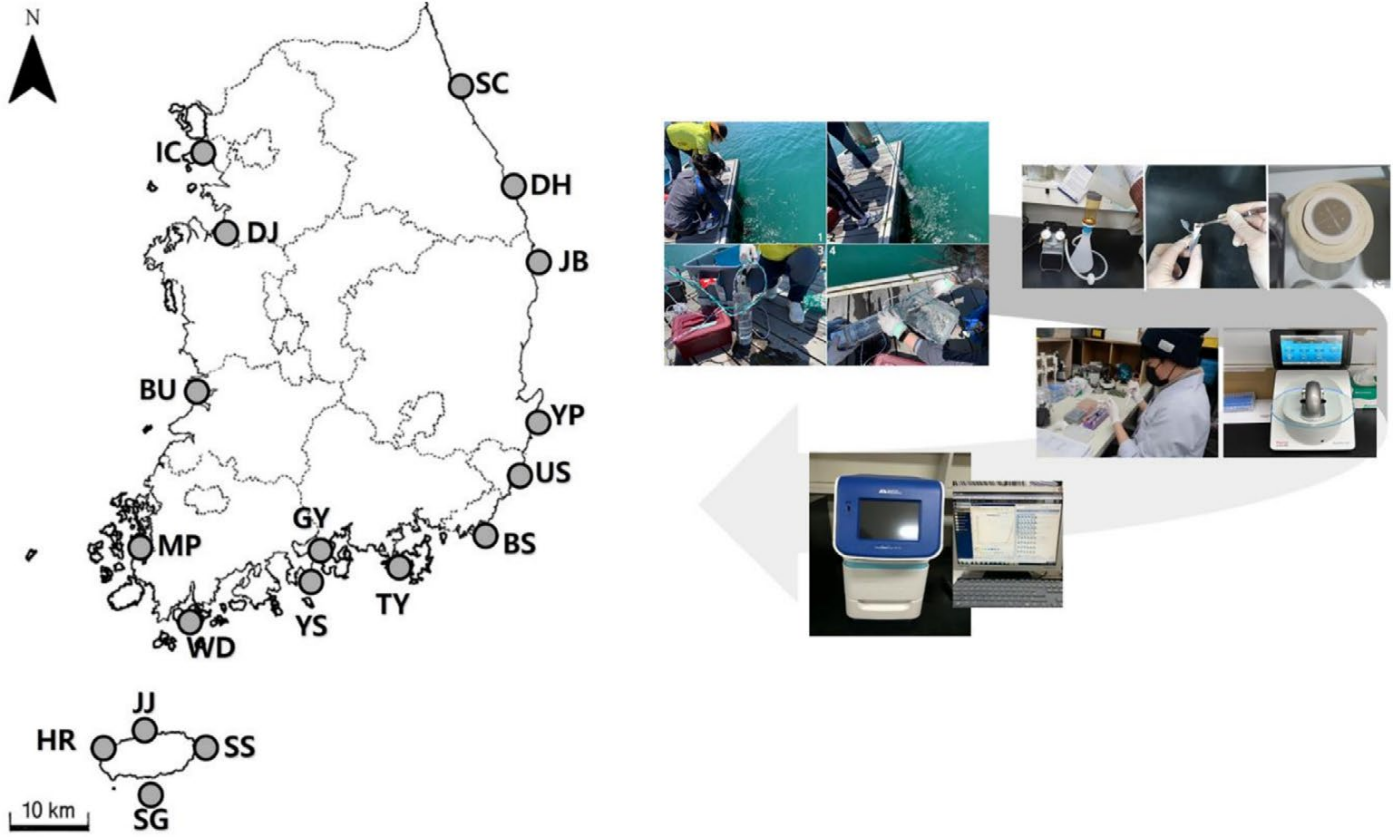
Nationwide harbor monitoring workflow and sampling locations. Map of 18 nationwide sampling ports and flow chart for quantitative analysis(qPCR) using eDNA.

Seawater samples were collected from 18 major harbors across the Korean coastline during spring (April), summer (July), and autumn (October) between 2019 and 2021 (Figure 2). At each harbor, two sub–sites were selected within the same region to maximize spatial coverage. From each sub–site, 8 L of seawater was collected using a 1.5 L water sampler (Table S1). To maintain sample integrity, collected water was stored at temperatures below 10°C until filtration. Filtration was performed within 12 h of collection using an Electrical Aspirator (JEIO TECH, Daejeon, Korea). Samples were pre–filtered through 300 μm nylon mesh to remove large debris and planktonic organisms, then passed through 3.0 μm mixed cellulose ester (MCE) membranes (ADVANTEC, Tokyo, Japan). Filters were placed into sterile 1.5 mL microcentrifuge tubes, immediately frozen on dry ice, and transported to the laboratory for DNA extraction.

### 2.2 DNA Extraction and Species–Specific qPCR Assay

Genomic DNA captured on MCE membranes (Aurora Pro Scientific, New Jersey, USA) during water filtration was extracted using the DNeasy Blood and Tissue Kit (Qiagen, Germany), following the manufacturer's protocol with minor modifications to maximize eDNA yield. DNA was eluted in 100 μL of AE buffer. DNA concentration and purity were assessed using a NanoDrop OneC spectrophotometer (Thermo Fisher Scientific, Madison, USA) and stored at −20°C until use.

The primers and probe used in this study were derived from our patented technology (KR 10–2023–0000688) specifically developed to detect only *A. aspersa* (Forward primer: 5′–TTATATTGATTATTTCTTCCA–3′; Reverse primer: 5′–GAACCGAGAATACTAGAAACA–3′; Probe: 5′–CCCGGCTGTTGATTGTGCAA–3′ [FAM/BHQ1]). These oligonucleotides were validated both in silico (NCBI BLAST) and in vitro against DNA from 128 non‐target taxa, including native ascidians ( *Styela clava* Herdman, 1881 and *S. plicata* (Lesueur, 1823)), ensuring highly specific amplification of *A. aspersa* DNA. Detailed oligonucleotide sequences and assay performance metrics are provided in Table S2.

Quantitative PCR (qPCR) reactions were performed in 20 μL reaction volumes containing 10 μL of qPCRBIO Probe Mix HiROX (PCR Biosystems, UK), 5 μL of eDNA template, 1 μL of TaqMan probe (5 pmol), 1 μL each of forward and reverse primers (10 pmol), and 2 μL of DEPC‐treated water. Thermal cycling was carried out on an Applied Biosystems StepOnePlus Real‐Time PCR System (Thermo Fisher Scientific) under the following conditions: initial denaturation at 95°C for 3 min, followed by 45 cycles of 95°C for 5 s and 60°C for 30 s, with a final extension at 72°C for 7 min.

### 2.3 Assay Performance, Limit of Detection, and Quantification

A synthetic DNA standard containing the assay target sequence was serially diluted (1 ng to 1 fg) to generate a standard curve. The assay exhibited strong linearity (*R* ^2^ > 0.99) and an efficiency of 110.1%. The Limit of Detection (LOD) was determined as the lowest DNA concentration consistently amplified in ≥ 95% of replicates, whereas the Limit of Quantification (LOQ) was defined as the lowest DNA concentration quantified with a coefficient of variation of < 35%.

### 2.4 Data Analysis and Visualization

All analyses were performed on water samples Raw qPCR tables exported by StepOnePlus were normalized (the standard curve is shown in Figure S4). Only numeric Ct entries explicitly reported as Ct (or unambiguously as Ct Mean) were retained; undetermined or non‐numeric fields were discarded. For each harbor, Ct distributions were summarized using medians, ranges, and IQRs, and visualized as boxplots. To compare detection patterns across years (2019–2022), sites were matched where metadata allowed. We summarized cycle threshold (Ct) values from the species‐specific qPCR assay using descriptive statistics (median, interquartile range [IQR], and range) and produced boxplots by harbor and season. And then, settlement‐plate percent cover for *A. aspersa* was compiled for the same sites and compared against eDNA Ct values to assess concordance (lower Ct ≈ higher cover); if identical sites could not be compared due to investigation changes in survey coverage, we restricted comparisons to representative harbors from the East Sea, West Sea, South Sea, and Jeju Sea to preserve geographic balance. Ct values were summarized by harbor (BU, TY, YP, JJ) using median, interquartile range (IQR), and minimum–maximum. No formal statistical tests (e.g., ANOVA) were performed due to dataset limitations. Only harbors consistently sampled in 2022 were included.

## 3 Results

### 3.1 Assay Performance

The assay reliably detected *A. aspersa* DNA at low concentrations, with clear amplification curves even below 10 copies per reaction. Negative controls remained consistently undetected across all experimental runs, and positive controls consistently produced amplification, confirming the validity and repeatability of the assay. Table 1 summarizes these results, showing the lowest detected DNA concentrations (LOD), limit of quantification (LOQ), and standard amplification efficiency.

**TABLE 1:** Summary of qPCR assay performance characteristics for detection of *Ascidiella aspersa* (KR1020230000688).

Assay target	LOD 95% (copies/reaction)	LOQ (copies/reaction)	Ct mean (±SD)	ΔRn threshold	Detection range	*N* (replicates)
*Ascidiella aspersa* (KR1020230000688)	10	25	2.76 ± 4.83	0.02	10–10^5^	33

*Note:* LOD 95% and LOQ were estimated based on standard dilution series. Ct values are from environmental sample testing across seasons and sites. ΔRn threshold refers to the amplification threshold used for detection.

The species‐specific qPCR assay demonstrated high analytical performance and strong specificity, showing no amplification of non‐target species, including other ascidians commonly present in Korean coastal habitats. Standard curves generated from serial dilutions of synthetic *A. aspersa* DNA showed excellent linearity (*R* ^2^ = 0.9962) and amplification efficiency (110.1%). The assay's limit of detection (LOD) was determined as 12 copies/reaction, and the limit of quantification (LOQ) as 598 copies/reaction, indicating that even trace concentrations of *A. aspersa* DNA can be reliably detected in environmental samples. Negative control replicates consistently showed no amplification, confirming robust contamination control. The patented species‐specific qPCR assay (KR 10–2023–0000688) demonstrated high specificity and sensitivity under laboratory conditions. No amplification occurred in any of the non‐target species tested, indicating excellent discriminatory power.

### 3.2 Seasonal Patterns

**FIGURE 3:**
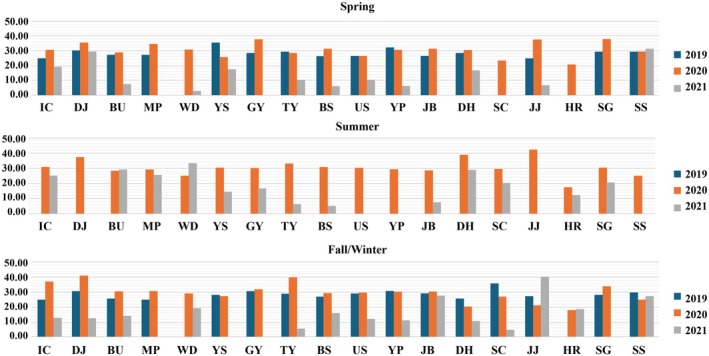
Seasonal variation in *Ascidiella aspersa* eDNA detection rate across Korean harbors in 2019–2022. Bars show the proportion of qPCR–positive site × samples per season (spring, summer, autumn). Detection is defined as amplification in ≥ 1 of the technical replicates with Ct ≤ 40 after baseline correction; non–detects are those with no amplification across replicates.

A total of 324 eDNA samples collected from 18 harbors were successfully amplified using the species‐specific *A. aspersa* qPCR assay. Detection frequency showed apparent seasonal differences: summer exhibited the highest detection rate (72%, *n* = 78/108 samples), followed by spring (48%, *n* = 52/108) and autumn (20%, *n* = 22/108). Mean Ct values also reflected this trend, being lower in summer (15.2 ± 2.1) compared to spring (23.5 ± 2.8) and autumn (29.8 ± 3.5). These seasonal differences were visually evident during 2019–2022, but no formal statistical testing was performed at this stage (Figure 3). This pattern is likely linked to biological processes: summer corresponds to the primary reproductive and settlement period of *A. aspersa* , when spawning and larval release increase the amount of organismal DNA, while warmer water may enhance DNA shedding rates and dispersal.

**FIGURE 4:**
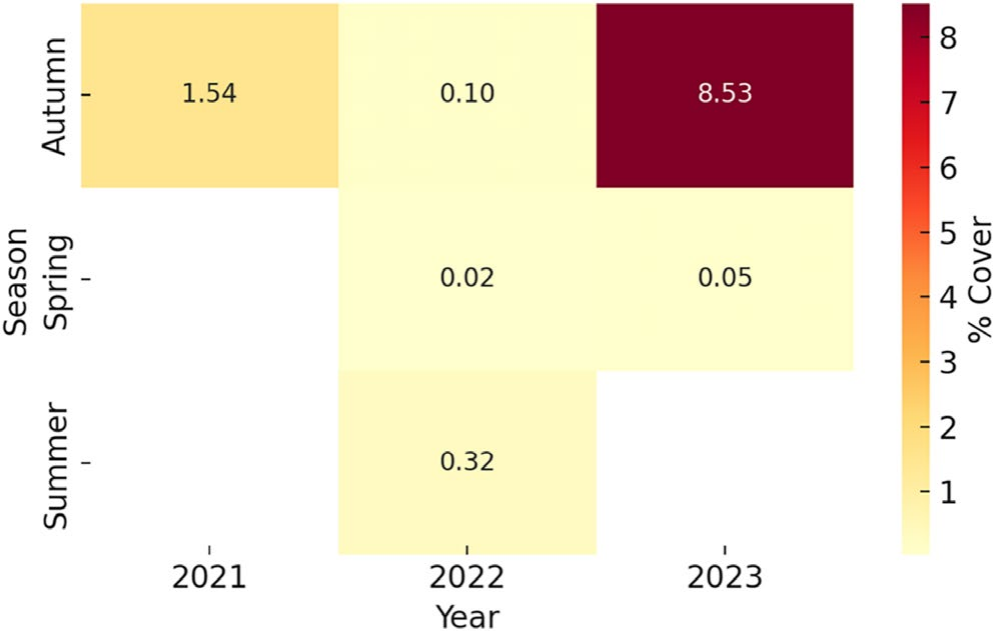
Seasonal distribution of qPCR Ct values for *Ascidiella aspersa* across Korean harbors in 2022. The heatmap shows lower Ct values (higher eDNA concentrations) during summer months, consistent with seasonal proliferation likely driven by elevated seawater temperatures and reproductive activity.

Across the 3‐year observation period, *A. aspersa* exhibited a recurring seasonal pattern characterized by peak percent cover during summer months. This trend, shown in Figure 4, likely reflects enhanced reproductive activity and favorable environmental conditions such as elevated seawater temperature, supporting population surges during warmer periods.

Ct distributions indicated measurable differences between harbors in summer 2022. BU (Bieung; *N* = 14) showed a median Ct of 14.25 (IQR = 17.71; range = 3.18–42.08), whereas TY (Tongyeong; *N* = 56) showed a median Ct of 28.00 (IQR = 29.00; range = 2.00–44.00). Differences were apparent, but statistical significance was not assessed. Seasonal Ct distributions and detection frequencies are illustrated in Figures 3 and 4, with site‐specific seasonal detection rates summarized in Table 2.

**TABLE 2:** Summary of qPCR Ct values for *Ascidiella aspersa* (summer 2022).

Harbor	N	Median	IQR	Range (min–max)
BU (Bieung)	14	14.25	17.71	3.18–42.08
TY (Tongyeong)	56	28.00	29.00	2.00–44.00

**FIGURE 5:**
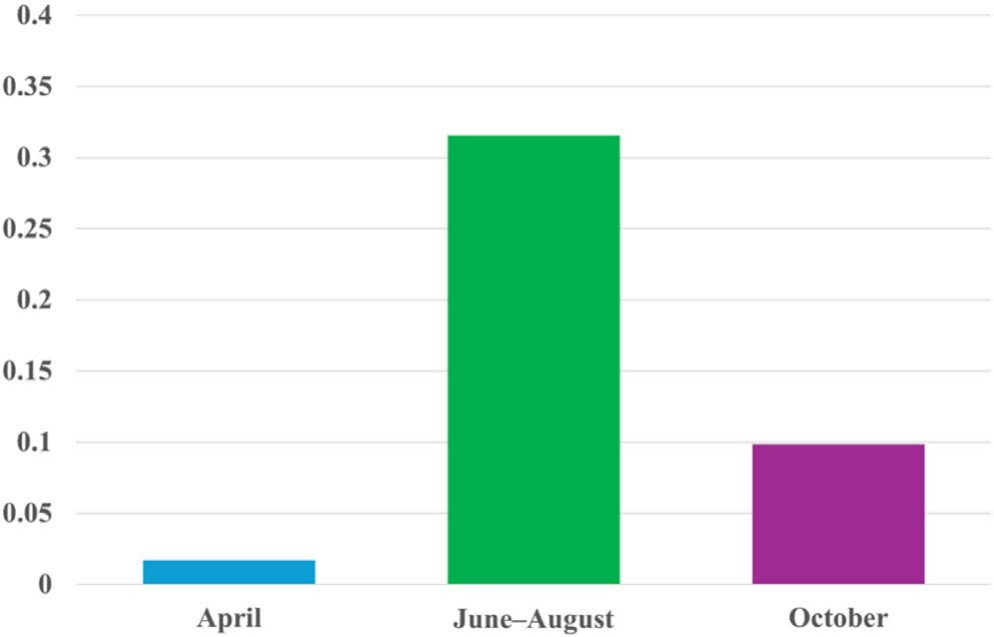
Seasonal variation in the percent cover of *Ascidiella aspersa* at Korean coastal sites during 2022. Mean percentage cover values are shown for spring (April), summer (June–August), and autumn (October) based on visual assessment surveys. Notably higher values were observed during summer, suggesting seasonal proliferation of *A. aspersa* .

Additionally, we summarized *A. aspersa* detection abundance in BU (Bieung) and TY (Tongyeong) during the summer of 2022 using currently available eDNA datasets. Ct distributions showed site‐level differences consistent with field observations, but statistical testing was not performed due to limited sample reproducibility. Sedimentation‐plate surveys at the same sites showed measurable *A. aspersa* cover, consistent with the eDNA signal (see Tables S3–S5; Figures S1–S3, S5). As shown in Figure 5, the seasonal variation in percent cover during 2022 indicates a notable increase in *A. aspersa* coverage during summer. The visual assessment revealed significantly higher percent cover in June–August compared to spring (April) and autumn (October), supporting the hypothesis of temperature‐associated bloom periods.

**FIGURE 6:**
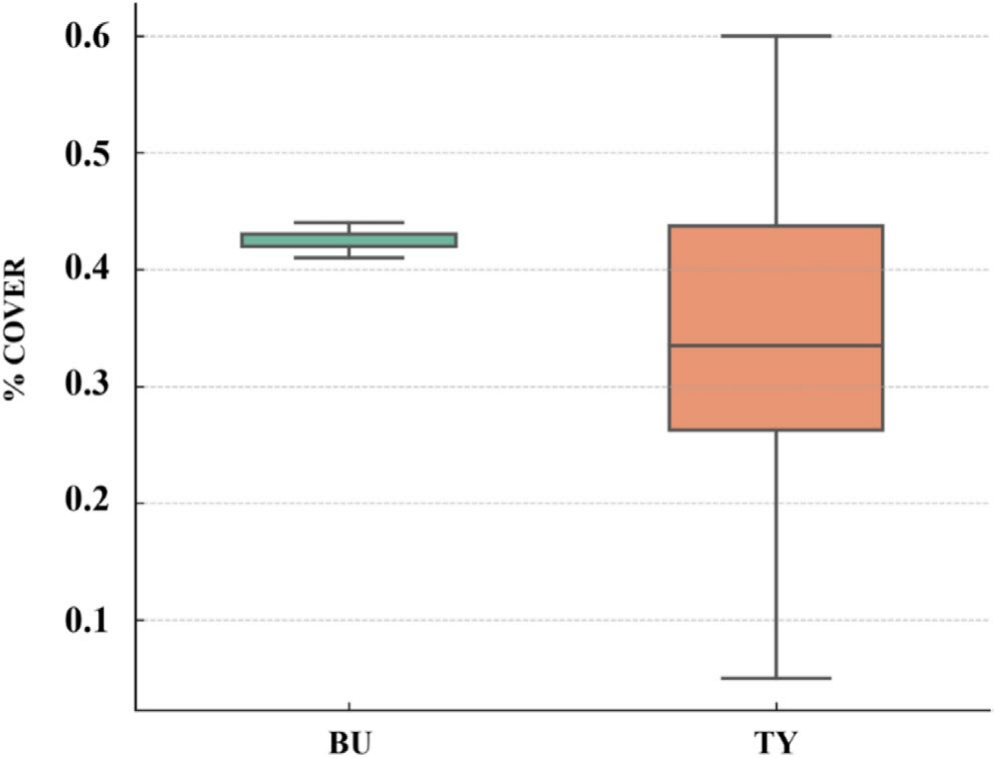
Comparison of percent cover of *Ascidiella aspersa* between Bieung (BU) and Tongyeong (TY) in 2022. Boxplot shows the site‐specific variability in percent cover *of A. aspersa*. Bieung (BU) exhibited consistently high coverage with low variability, while Tongyeong (TY) showed a broader range of values, indicating more spatial heterogeneity.

Analysis of the 2022 coverage rates in Bieung (BU) and Tongyeong (TY) revealed that BU had a higher average coverage rate with relatively stable values, while TY showed a wider range of variability, indicating site‐specific differences in settlement dynamics and the influence of local environmental factors (Figure 6).

Detailed qPCR results from surveys conducted at four representative harbors (BU, TY, YP, and JJ) in 2022 are summarized in Table 3. This table provides measured cycle threshold (Ct) values, detection outcomes, and the number of technical replicates analyzed for each sampling site, offering essential data that support the seasonal detection patterns described above. Positive detection was defined as amplification occurring in at least one of three technical replicates with a Ct value of ≤ 40, following MIQE guidelines (Bustin et al. 2009) and widely accepted practices in eDNA qPCR analyses (Goldberg et al. 2016; Harper et al. 2019). This threshold was applied consistently across all samples, and the detection results presented in Table 3 reflect outcomes obtained under this standardized criterion. Notably, several sites (e.g., BU—April 2022; JJ—April 2022; BU—October 2022; TY—October 2022) exhibited amplification signals despite low abundance, highlighting the high sensitivity of the assay for detecting trace levels of eDNA even when target concentrations were minimal.

**TABLE 3:** Species‐specific eDNA qPCR results for *Ascidiella aspersa* obtained from water samples collected at four representative harbors (BU, TY, YP, and JJ) in 2022.

Harbor	Region code	Date	Season	Ct	Replicate	Detection (0/1)	Assay version
Bieung	BU	2022.4.	Spring	18.1968	2	1	KR 10–2023–0000688
Tongyeong	TY	2022.4.	Spring	20.4941	2	1	
Yangpo	YP	2022.4.	Spring	32.2423	2	1	
Jeju	JJ	2022.4.	Spring	5.89625	2	1	
Bieung	BU	2022.7.	Summer	19.2638	2	1	
Tongyeong	TY	2022.7.	Summer	35.0671	2	1	
Yangpo	YP	2022.7.	Summer	37.6704	2	1	
Jeju	JJ	2022.7.	Summer	10.2228	2	1	
Bieung	BU	2022.10.	Autumn	26.1366	2	1	
Tongyeong	TY	2022.10.	Autumn	35.5924	2	1	
Yangpo	YP	2022.10.	Autumn	31.4016	2	1	
Jeju	JJ	2022.10.	Autumn	—	2	—	

*Note:* The table includes measured cycle threshold (Ct) values and detection outcomes for each sampling event, along with the number of technical replicates. Detection results are expressed as binary outcomes: 1 = detected (Ct ≤ 40 in ≥ 1 technical replicate), 0 = not detected (no amplification within 40 cycles). “—” indicates no amplification observed.

### 3.3 Geographic Expansion

**FIGURE 7:**
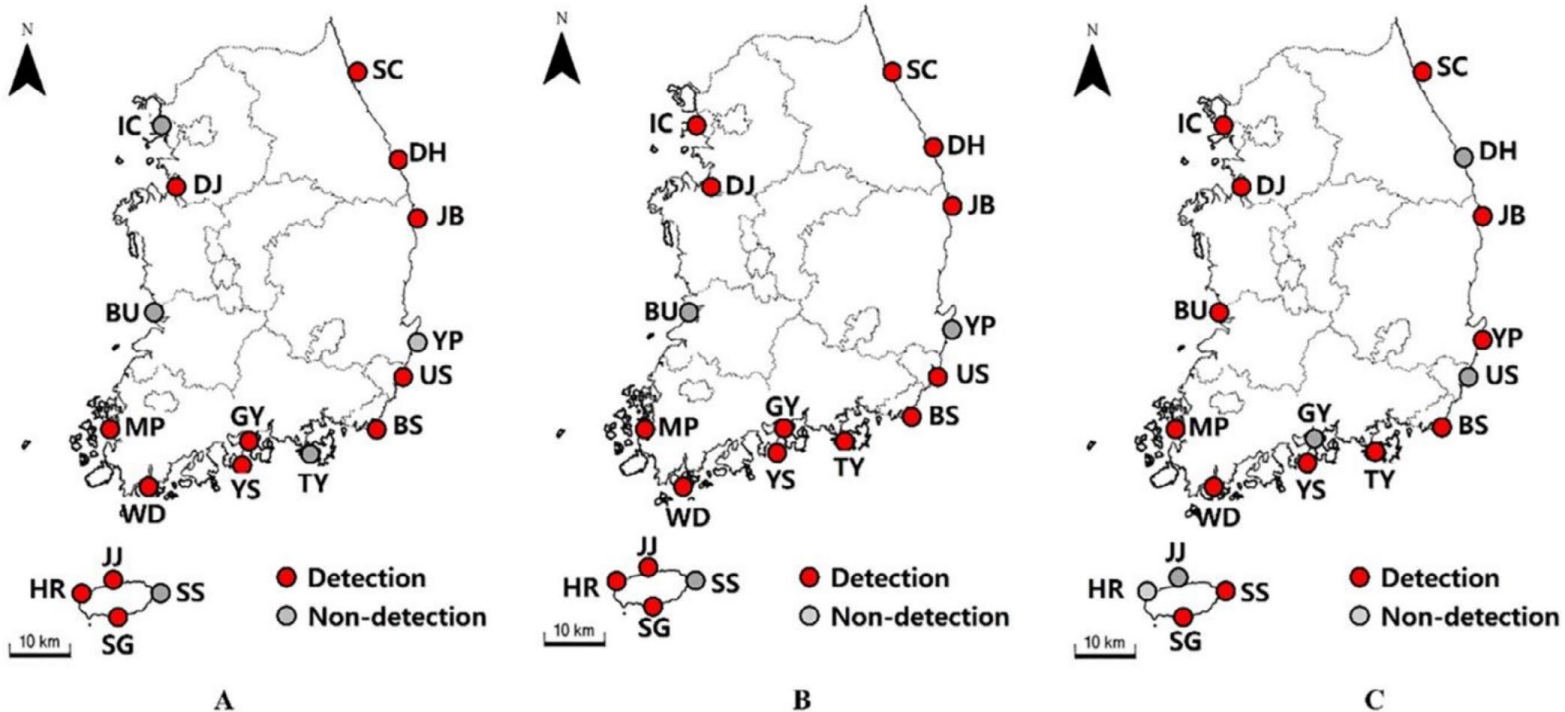
Geographic expansion of *Ascidiella aspersa* . Seasonal detection patterns of *A. aspersa* across 18 Korean coastal ports based on qPCR assay results from 2019 to 2022. Each panel (A: April, B: July, C: October) displays detection status of *A. aspersa* using environmental DNA (eDNA) analysis. Red circles indicate positive detections, and gray circles represent non‐detections.

Historical records indicated that *A. aspersa* was initially restricted to southern ports such as Tongyeong and adjacent harbors (Pyo et al. 2012). Our 3‐year dataset (2019–2021) revealed progressive geographic expansion, including first detections in Jeju Island (85% sample positivity, mean Ct = 18.0) and confirmed detection in other ports previously unreported by traditional surveys. Detection intensity was greatest in harbors with dense artificial structures and high shipping traffic, indicating anthropogenic vectors, including hull fouling and ballast water, as likely drivers of spread. Spatial changes in detection intensity and the emergence of newly positive sites are mapped in Figure 7.

### 3.4 Integration With Nationwide Monitoring

The nationwide harbor monitoring program using our patented qPCR assay provided conclusive evidence of the widespread presence of *A. aspersa* across Korean ports. Over 324 samples collected from 18 harbors confirmed previously known populations and identified new occurrences, including sites lacking prior visual records of the species. This combined molecular–traditional approach enhanced early detection capability and improved assessment of the species' distributional extent at a national scale, underscoring the utility of integrating eDNA diagnostics into routine biosecurity monitoring (Figure 7). The integration of molecular data with nationwide coverage represents a scalable model for early detection and rapid response (EDRR) and supports informed management interventions to minimize ecological and economic impacts of invasive ascidians.

## 4 Discussion

This study presents the first nationwide application of a species‐specific qPCR assay for the detection of the invasive solitary ascidian *A. aspersa* using environmental DNA (eDNA) in Korean waters. The patented assay (KR 10–2023–0000688) demonstrated high specificity and sensitivity, with no amplification of nontarget species and reliable detection of trace DNA concentrations. These characteristics are particularly valuable for the early detection of invasive species, which often occur at low abundance or in cryptic habitats where traditional survey methods can be ineffective.

The invasion history, spatial expansion, and genetic structure of *A. aspersa* in Korean coastal waters demonstrate a striking trajectory of ecological establishment over the past decade. The earliest comprehensive record of this species in Korea was presented by Pyo et al. (2012), who identified *A. aspersa* as a newly introduced ascidian based on morphological traits and phylogenetic analyses using partial 18S rDNA and mitochondrial COI sequences. Their survey documented the presence of the species in numerous harbors along the East Sea, Korea Strait, and Yellow Sea, revealing its capacity for rapid colonization of artificial substrates. Notably, however, Jeju Island remained uncolonized during this initial phase, indicating that the species was still in the process of expanding its range. Building upon this foundational knowledge, Lee et al. (2023) investigated the genetic diversity and population structure of *A. aspersa* across both Korean and global populations. Using an expanded dataset of 154 mt‐COI and 127 18S rDNA sequences—including 80 mt‐COI and 79 18S sequences from Korean waters—the study identified 14 distinct mt‐COI haplotypes in Korea. This diversity points to the likelihood of multiple introduction events or subsequent secondary dispersal following initial establishment. The dominant haplotype (Hap_3), present across all sampled locations, reflects strong genetic connectivity among Korean populations, whereas the presence of several site‐specific haplotypes suggests localized diversification and potential adaptive responses to distinct environmental conditions.

The contrast between these two studies illustrates how our understanding of *A. aspersa* invasion dynamics in Korea has evolved from initial detection and morphological characterization (Pyo et al. 2012) to detailed molecular insights into population structure (Lee et al. 2023). Whereas the earlier report captured the species during its nascent colonization stage (2009–2011), when its distribution remained spatially constrained and excluded Jeju Island, the later work documented a genetically diverse and well‐established population by 2021 that had become widely distributed along the Korean coastline, including Jeju. This progression reflects a clear ecological transition from the early invasion phase—marked by initial establishment and range expansion—to a saturation phase, characterized by stable, widely distributed populations occupying a variety of available habitats.

The present eDNA‐based qPCR survey further extends this understanding by enabling sensitive detection and fine‐scale monitoring of *A. aspersa* across space and time. Targeting the mitochondrial COI region with species‐specific primers and probes, the assay achieved high analytical performance, reliably detecting as few as 12 DNA copies per reaction and exhibiting no cross‐reactivity with 128 non‐target taxa. Applied to more than 300 seawater samples collected from 18 harbors between 2019 and 2022, the assay revealed pronounced seasonal variation in eDNA concentration, with detection peaks during summer coinciding with the species' reproductive period. Importantly, *A. aspersa* was also detected in sites where its presence had not been previously reported, including Jeju Island and several eastern ports. These findings not only confirm the species' broader contemporary distribution but also underscore the value of eDNA‐based diagnostics as a powerful tool for detecting overlooked or emerging populations that might otherwise evade conventional survey methods.

The seasonal variation in detection signals, with the highest DNA concentrations in summer and reduced signals in spring and autumn, is consistent with the reproductive ecology of *A. aspersa* . Spawning and larval release during summer likely increase the amount of organismal and extra‐organismal DNA in surrounding waters. Although no formal statistical testing was performed, the seasonal differences were visually apparent, suggesting that summer may be the optimal window for eDNA‐based monitoring of this species.

The geographic distribution results confirmed not only previously reported populations in southern ports but also detections in Jeju Island and other harbors where prior visual records were absent. These findings suggest a broader and possibly continuing spread of *A. aspersa* in Korean coastal waters. The pattern of occurrence aligns with anthropogenic dispersal pathways, including shipping activities and the presence of artificial structures, both of which are known to facilitate ascidian establishment and secondary spread.

Our study confirmed the concordance between eDNA and plate surveys. The species‐specific qPCR assay produced eDNA patterns that tracked settlement‐plate observations: harbors with lower Ct values tended to exhibit higher *A. aspersa* cover. BU and TY emerged as hotspots consistent with rapid establishment on artificial substrates. While spatial coverage varied among years (due to program logistics), the present analysis focused on summer 2022 water samples demonstrates that the patented assay (KR 10–2023–0000688) yields decision‐relevant signals for early detection and prioritization. Future expansions in temporal/seasonal replication and multi‐harbor coverage will enable formal hypothesis testing and stronger inference. This pattern is further visualized in Figure S6, where lower Ct values corresponded to higher settlement cover across matched harbor‐season combinations.

According to Rodriguez‐Ezpeleta et al. (2021), this assay detects both organismal eDNA (e.g., larvae, eggs, intact cells) and extra‐organismal eDNA (e.g., mucus, feces, degraded tissue). As such, positive signals indicate the presence of *A. aspersa* DNA but not necessarily live adults. However, repeated detections across multiple seasons and spatially distinct harbors strongly suggest established populations. The integration of a species‐specific molecular assay into a nationwide harbor monitoring program demonstrates how eDNA diagnostics can complement or even enhance traditional monitoring. This approach allows for early detection, supports risk assessment, and provides actionable data for biosecurity and management frameworks. While this study focused on one target species, the methodology can be adapted to other invasive species of concern, supporting scalable and rapid‐response monitoring systems. Advancements in eDNA detection technologies are continuing to expand the scope and responsiveness of molecular biomonitoring. In particular, CRISPR/Cas12a‐based platforms such as SHERLOCK, DETECTR, and SENTINEL are emerging as promising alternatives to qPCR, offering rapid, highly specific, and field‐deployable detection of target DNA (Chen et al. 2018; Fozouni et al. 2021; Kellner et al. 2019; Yu et al. 2022). Although not applied in the present study, these approaches represent the next frontier in bio‐surveillance and may be integrated into future aquatic invasive species monitoring frameworks.

## 5 Conclusion

We present the first species‐specific qPCR assay for *A. aspersa* with high sensitivity and specificity. Applied to environmental samples, the assay revealed clear seasonal and spatial patterns consistent with ongoing invasion and range expansion in Korean waters. This patented molecular tool provides an effective platform for early detection, risk assessment, and management of invasive marine species.

## Author Contributions

**Jeounghee Lee:** conceptualization (lead), data curation (lead), formal analysis (lead), funding acquisition (equal), investigation (equal), methodology (lead), project administration (lead), resources (lead), software (lead), supervision (equal), validation (equal), visualization (equal), writing – original draft (lead), writing – review and editing (lead). **Seongjae Kim:** formal analysis (supporting), investigation (equal), methodology (equal), resources (equal). **Soyeon Kwon:** data curation (supporting), formal analysis (supporting), investigation (equal), methodology (equal), resources (supporting). **Jinho Lee:** formal analysis (supporting), investigation (equal), methodology (supporting), resources (supporting). **Sook Shin:** conceptualization (equal), project administration (lead), supervision (equal).

## Conflicts of Interest

The authors declare no conflicts of interest.

## Supporting information


**Data S1:** ece372453‐sup‐0001‐Supinfo01.docx.

## Data Availability

All data supporting the findings of this study are available within the article and its Supporting Information S1.
