# Supplementary material for: Development and Application of a Species–Specific eDNA–Based qPCR Assay for Early Detection of the Invasive Ascidian Ascidiella aspersa
Source: Ecol Evol. 2025 Nov 10;15(11):e72453. doi: 10.1002/ece3.72453 (PMC12600031; doi:10.1002/ece3.72453)
Supplement: Supplementary file 1 — Data S1: ece372453‐sup‐0001‐Supinfo01.docx. [file ECE3-15-e72453-s001.docx]

Supplementary Materials

This file contains all supplementary tables and figures supporting the manuscript.

**Table S1. Sampling metadata of 18 Korean harbors (2019–2023): site names and GPS coordinates as in the main manuscript.**

| **Name of site** | **GPS** | |
| --- | --- | --- |
|  | **Pont 1** | **Pont 2** |
| Incheon(IC) | 37.4609763 126.6151999 | 37.4582334 126.6246186 |
| Dangjin(DJ) | 36.9873421 126.7462130. | 36.9866793 126.7457836 |
| Bieung (BU) | 35.9361879 126.5278411 | 35.9363492 126.5280674 |
| Mokpo(MK) | 34.7828131 126.3882664 | 34.7833947 126.3889505 |
| Wando(WD) | 34.3181425 126.7671018 | 34.3177159 126.7672770 |
| Yeosu(YS) | 34.7422872 127.7554700 | 34.7420605 127.7553363 |
| Gwangyang(GY) | 34.8949166 127.6578629 | 34.8935399 127.6557575 |
| Tongyeong(TY) | 34.8274753 128.4351877 | 34.8273333 128.4346863 |
| Busan(BS) | 35.0939519 129.0956069, | 35.1007218 129.0944471 |
| Ulsan(US) | 35.5188911 129.3753223 | 35.5206216 129.3754880 |
| Yangpo(YP) | 35.8781379 129.5195257 | 35.8779264 129.5195958 |
| Juckbeun(JB) | 37.0551032 129.4175351 | 37.0557160 129.4183563 |
| Donghae(DH) | 37.4876817 129.1269807 | 37.4894419 129.1254697 |
| Sokcho(SC) | 38.2106103 128.5965584 | 38.1956918 128.5934751 |
| Jeju(JJ) | 33.5206669 126.5320612 | 33.5191244 126.5290630 |
| Hanrim(HR) | 33.4199904 126.2608526, | 33.4196568 126.2606864 |
| Seogwipo(SG) | 33.2403087 126.5592530 | 33.2409402 126.5595186 |
| Sungsan(SS) | 33.4723635 126.9292313 | 33.4722050 126.9257933 |

**Table S2. Detailed oligonucleotide sequences and assay performance metrics for the species-specific qPCR assay developed for *Ascidiella aspersa***. Primer and probe sequences, amplicon sizes, and analytical performance parameters (slope, R², efficiency, and LOD/LOQ) are provided. Specificity validation was conducted against 128 non-target taxa, including native ascidians (*Styela clava and S. plicata*).

| **Name** | **Sequence (5’-3’)** |
| --- | --- |
| **Forward primer** | 5′–TTATATTGATTATTTCTTCCA–3′ |
| **Reverse primer** | 5′–GAACCGAGAATACTAGAAACA–3′ |
| **Probe** | 5′–CCCGGCTGTTGATTGTGCAA–3′ |
| **Performance** | Efficiency 110.1%; R² 0.9962; LOD 12 copies/rxn; LOQ 598 copies/rxn |
| **Amplicon size** | 160bp |

**Table S3. Seasonal percent cover of *Ascidiella aspersa* in Bieung (BU) and Tongyeong (TY) during 2022.** Each row shows seasonal settlement plate percent cover. Data are presented as median, 25th percentile (q25), 75th percentile (q75), minimum, maximum, and sample size (n). When n=1, all values are identical.

| **Harbor** | **Season** | **n** | **median** | **q25** | **q75** | **min** | **max** |
| --- | --- | --- | --- | --- | --- | --- | --- |
| **Bieung (BU)** | Summer | 1 | 0.425 | 0.425 | 0.425 | 0.425 | 0.425 |
| **Bieung (BU)** | Autumn | 1 | 0.000 | 0.000 | 0.000 | 0.000 | 0.000 |
| **Tongyeong (TY)** | Summer | 1 | 0.610 | 0.610 | 0.610 | 0.610 | 0.610 |
| **Tongyeong (TY)** | Autumn | 1 | 0.051 | 0.051 | 0.051 | 0.051 | 0.051 |

**Table S4. Settlement-plate monitoring data for 4 harbors surveyed in 2021-2023.** Percent cover values represent the mean of 10 settlement plates per harbor. Occurrence (0/1) indicates presence or absence of *Ascidiella aspersa*.

| **Harbor** | **RegionCode** | **Year** | **Month** | **Season** | **PercentCover** | **Occurrence(0/1)** |
| --- | --- | --- | --- | --- | --- | --- |
| **Bieung** | (BU) | 2021 | 4 | Spring | 2.43 | 1 |
| **Bieung** | (BU) | 2021 | 7 | Summer | 21.9 | 1 |
| **Bieung** | (BU) | 2021 | 10 | Autumn | 1.19 | 1 |
| **Bieung** | (BU) | 2022 | 4 | Spring | 0.3 | 1 |
| **Bieung** | (BU) | 2022 | 7 | Summer | 0.42 | 1 |
| **Bieung** | (BU) | 2022 | 10 | Autumn | 0 | 0 |
| **Bieung** | (BU) | 2023 | 4 | Spring | 0.2 | 1 |
| **Bieung** | (BU) | 2023 | 7 | Summer | 0.01 | 1 |
| **Bieung** | (BU) | 2023 | 10 | Autumn | 0 | 0 |
| **Tongyeong** | (TY) | 2021 | 4 | Spring | 5.76 | 1 |
| **Tongyeong** | (TY) | 2021 | 7 | Summer | 16.25 | 1 |
| **Tongyeong** | (TY) | 2021 | 10 | Autumn | 0 | 0 |
| **Tongyeong** | (TY) | 2022 | 4 | Spring | 5.26 | 1 |
| **Tongyeong** | (TY) | 2022 | 7 | Summer | 0.61 | 1 |
| **Tongyeong** | (TY) | 2022 | 10 | Autumn | 0.05 | 1 |
| **Tongyeong** | (TY) | 2023 | 4 | Spring | 0.94 | 1 |
| **Tongyeong** | (TY) | 2023 | 7 | Summer | 1.41 | 1 |
| **Tongyeong** | (TY) | 2023 | 10 | Autumn | 0 | 0 |
| **Yangpo** | (YP) | 2021 | 4 | Spring | 1.12 | 1 |
| **Yangpo** | (YP) | 2021 | 7 | Summer | 2.37 | 1 |
| **Yangpo** | (YP) | 2021 | 10 | Autumn | 0 | 0 |
| **Yangpo** | (YP) | 2022 | 4 | Spring | 0.36 | 1 |
| **Yangpo** | (YP) | 2022 | 7 | Summer | 0.02 | 1 |
| **Yangpo** | (YP) | 2022 | 10 | Autumn | 0 | 0 |
| **Yangpo** | (YP) | 2023 | 4 | Spring | 0.52 | 1 |
| **Yangpo** | (YP) | 2023 | 7 | Summer | 0.66 | 1 |
| **Yangpo** | (YP) | 2023 | 10 | Autumn | 0 | 0 |
| **Jeju** | (JJ) | 2021 | 4 | Spring | 0 | 0 |
| **Jeju** | (JJ) | 2021 | 7 | Summer | 0 | 0 |
| **Jeju** | (JJ) | 2021 | 10 | Autumn | 0 | 0 |
| **Jeju** | (JJ) | 2022 | 4 | Spring | 0 | 0 |
| **Jeju** | (JJ) | 2022 | 7 | Summer | 0.01 | 1 |
| **Jeju** | (JJ) | 2022 | 10 | Autumn | 0 | 0 |
| **Jeju** | (JJ) | 2023 | 4 | Spring | 0 | 0 |
| **Jeju** | (JJ) | 2023 | 7 | Summer | 0 | 0 |
| **Jeju** | (JJ) | 2023 | 10 | Autumn | 0 | 0 |

| **Harbor** | **Season** | **n** | **mean** | **median** | **q25** | **q75** | **min** | **max** |
| --- | --- | --- | --- | --- | --- | --- | --- | --- |
| **Bieung** | Spring | 1 | 2.43 | 2.43 | 2.43 | 2.43 | 2.43 | 2.43 |
| **Bieung** | Summer |  |  |  |  |  |  |  |
| **Bieung** | Autumn |  |  |  |  |  |  |  |
| **Tongyeong** | Spring | 1 | 5.76 | 5.76 | 5.76 | 5.76 | 5.76 | 5.76 |
| **Tongyeong** | Summer |  |  |  |  |  |  |  |
| **Tongyeong** | Autumn |  |  |  |  |  |  |  |
| **Yangpo** | Spring | 1 | 1.12 | 1.12 | 1.12 | 1.12 | 1.12 | 1.12 |
| **Yangpo** | Summer |  |  |  |  |  |  |  |
| **Yangpo** | Autumn |  |  |  |  |  |  |  |
| **Jeju** | Spring | 1 | 0 | 0 | 0 | 0 | 0 | 0 |
| **Jeju** | Summer |  |  |  |  |  |  |  |
| **Jeju** | Autumn |  |  |  |  |  |  |  |

**Table S5. Summary statistics of settlement monitoring percent cover (2020–2022).** Summary of *Ascidiella aspersa* settlement monitoring data pooled by harbor (BU = Bieung, TY = Tongyeong, YP = Yangpo, JJ = Jeju) and season (Spring, Summer, Autumn) from 2020 to 2022. Values are shown as the number of observations (n), mean, median, 25th percentile (q25), 75th percentile (q75), minimum, and maximum percent cover (%). These statistics were used as the basis for Figure S1.


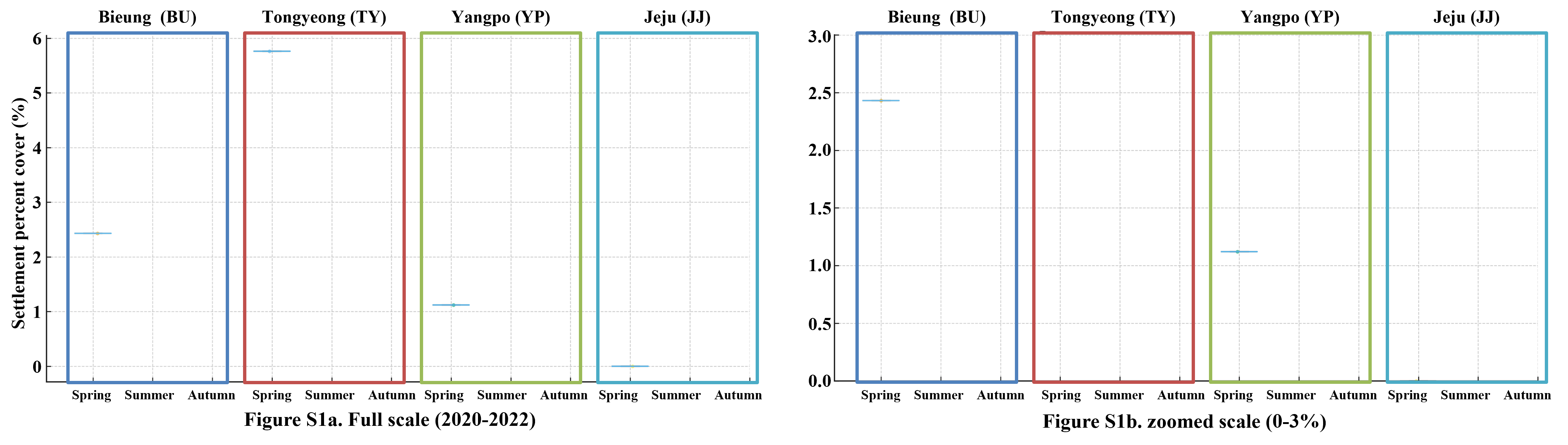


**Figure S1. Settlement monitoring (2020–2022): Box plots by harbor and season.** (a) Full-scale box plots (0–6%) showing seasonal settlement percent cover of Ascidiella aspersa across harbors (BU, TY, YP, JJ) from 2020 to 2022. (b) Zoomed box plots (0–3%) highlighting small values for improved visualization. Boxes indicate interquartile ranges, horizontal lines indicate medians, and dots represent outliers. Raw observations are plotted as jittered points.


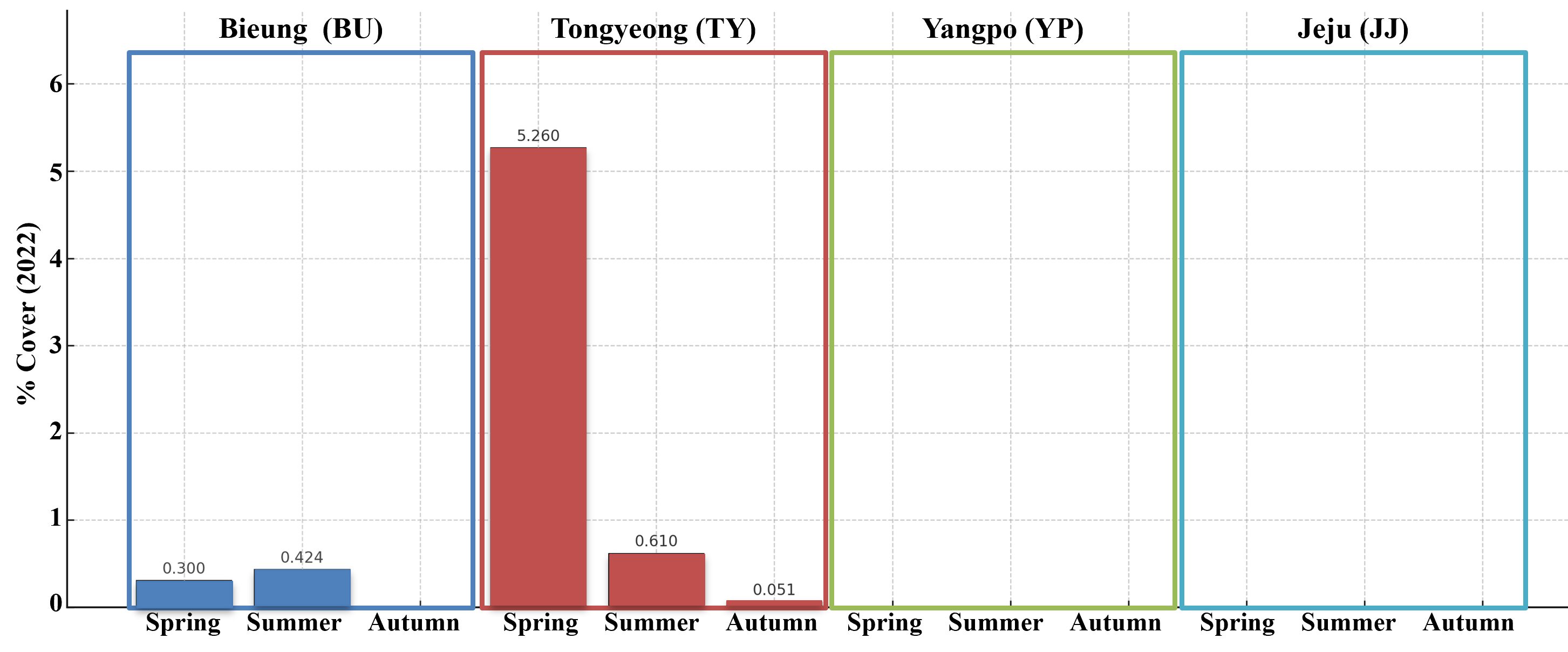


Figure S2. Seasonal percent cover of *Ascidiella aspersa* by Harbor×Season (2022). Settlement plate percent cover (%) for A. aspersa in Bieung (BU) and Tongyeong (TY) during 2022. Bars indicate seasonal percent cover; value labels show exact percentages (Spring, Summer, Autumn). The y-axis is restricted to highlight small differences among low values.


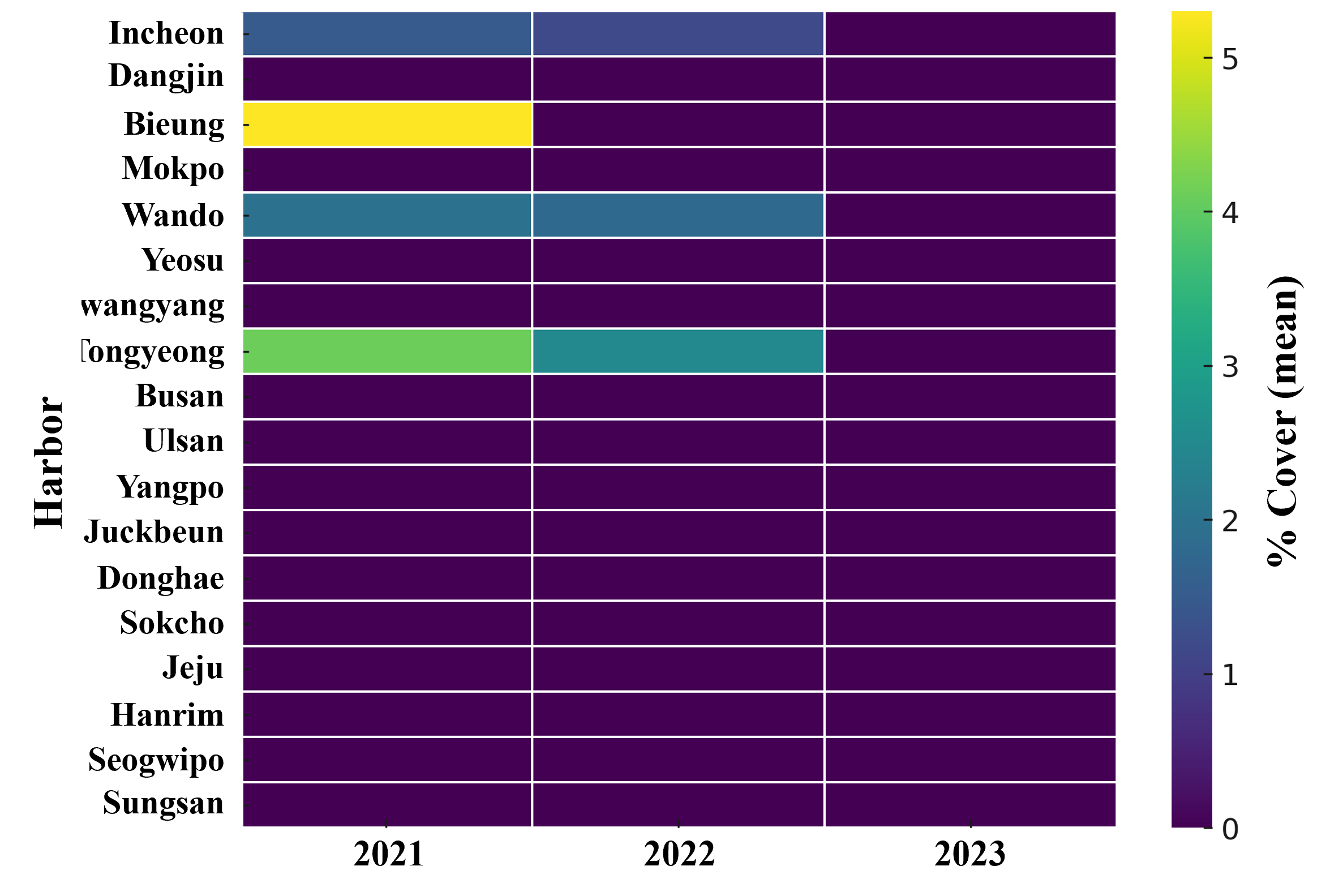


**Figure S3. Scatter plot of settlement percent cover by season.** Scatter plot showing seasonal variation in settlement cover across harbors. Summer values tended to be relatively higher, while spring and autumn values were mostly low or undetected.


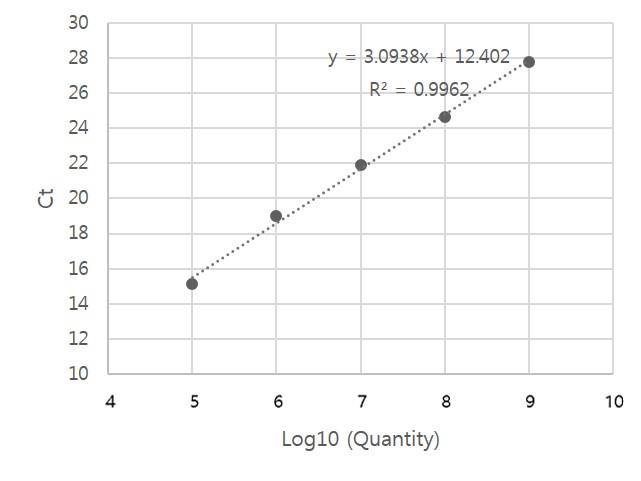


**Figure S4. Standard curve of qPCR assay for *Ascidiella aspersa.*** The relationship between Ct values and DNA concentration (log10 copies per reaction). R² and amplification efficiency values are provided. All assays showed highly linear standard curves.

**
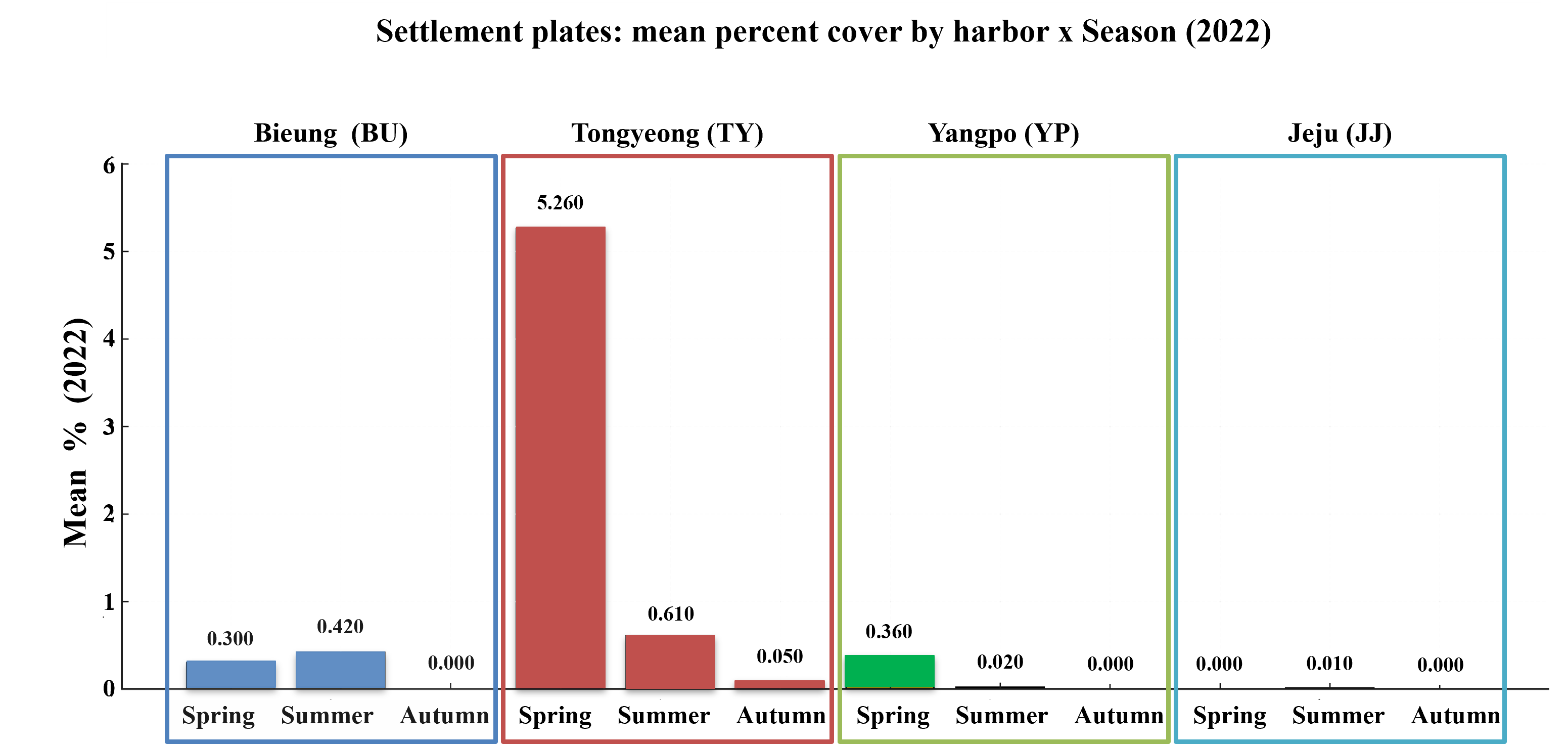
**

**Figure S5(a). Settlement plates: mean percent cover by Harbor×Season (2022).** Settlement monitoring results for 2022. Four harbors (Bieung, Tongyeong, Yangpo, Jeju) across three seasons (Spring, Summer, Autumn) are presented. Most combinations resulted in 0.000 (non-detections). Only Tongyeong in spring showed a relatively high mean cover of 5.260%. The y-axis was fixed to 0–6% to highlight small variations.

**
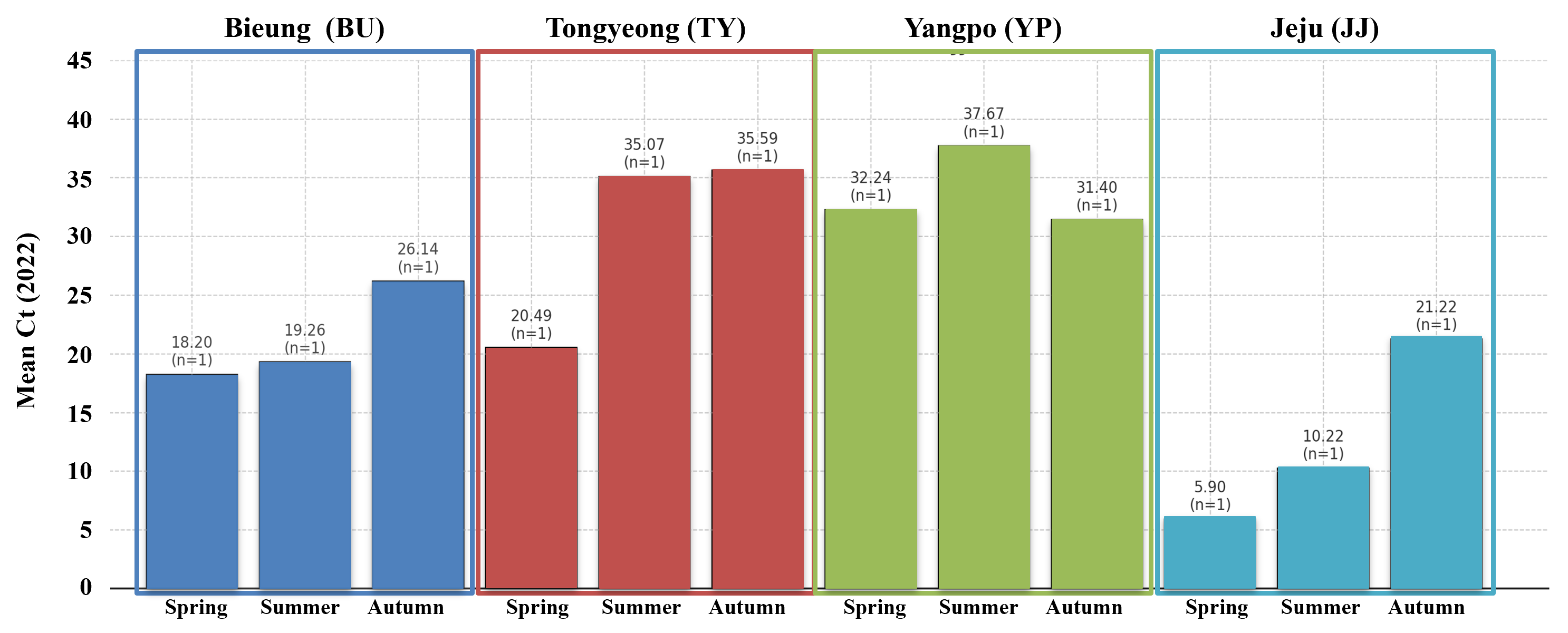
Figure S5(b1). eDNA qPCR monitoring in 2022: Mean Ct by Harbor×Season.** Bars represent mean Ct values for each harbor × season combination, with replicate numbers (n) shown above. Ct values were mostly within the 30–38 range. Lower Ct values (e.g., Jeju Spring=5.90, Jeju Summer=10.22) indicate higher DNA concentrations. Combinations with no data are shown as 0.00. The y-axis was fixed to 0–45 for comparability.

**
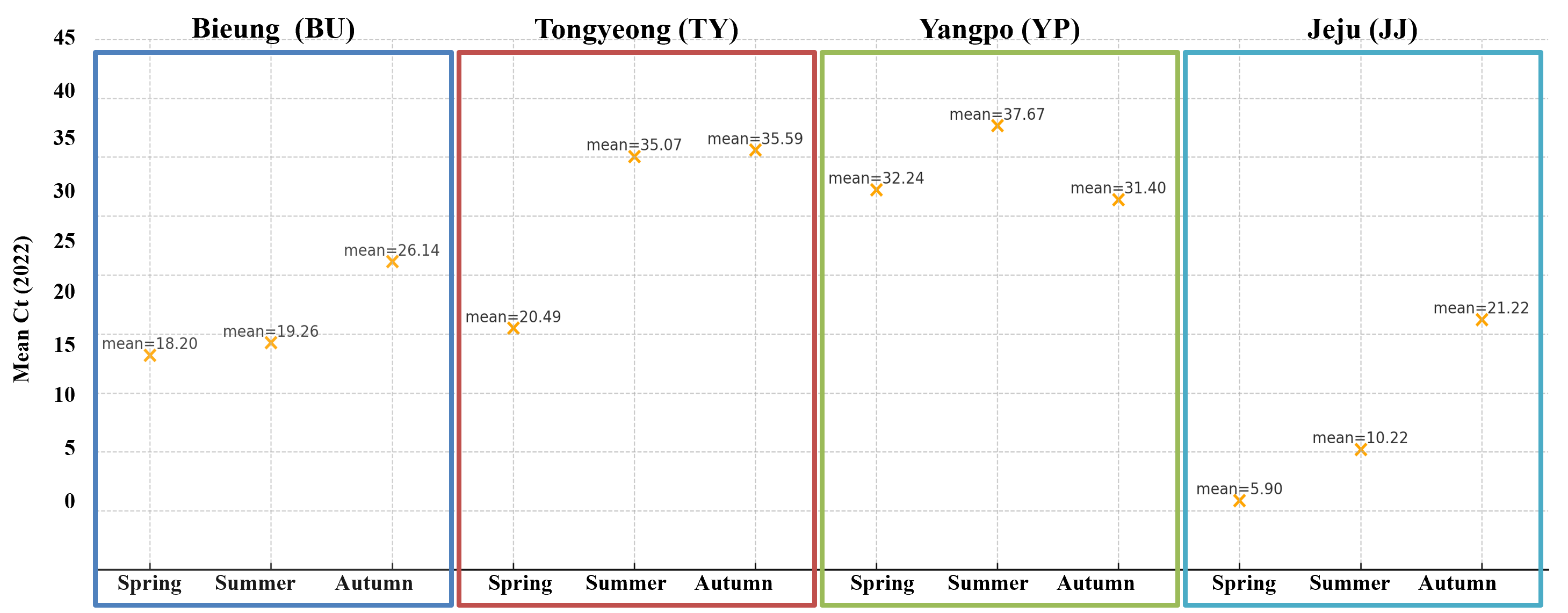
Figure S5(b2). eDNA qPCR monitoring in 2022: Ct replicate values by Harbor×Season.** Each dot represents a technical replicate. Text labels show mean Ct values for each combination. Data were unevenly distributed across harbors and seasons; notably, Jeju and Bieung exhibited low Ct values, confirming DNA detection. The y-axis was fixed to 0–45.

**
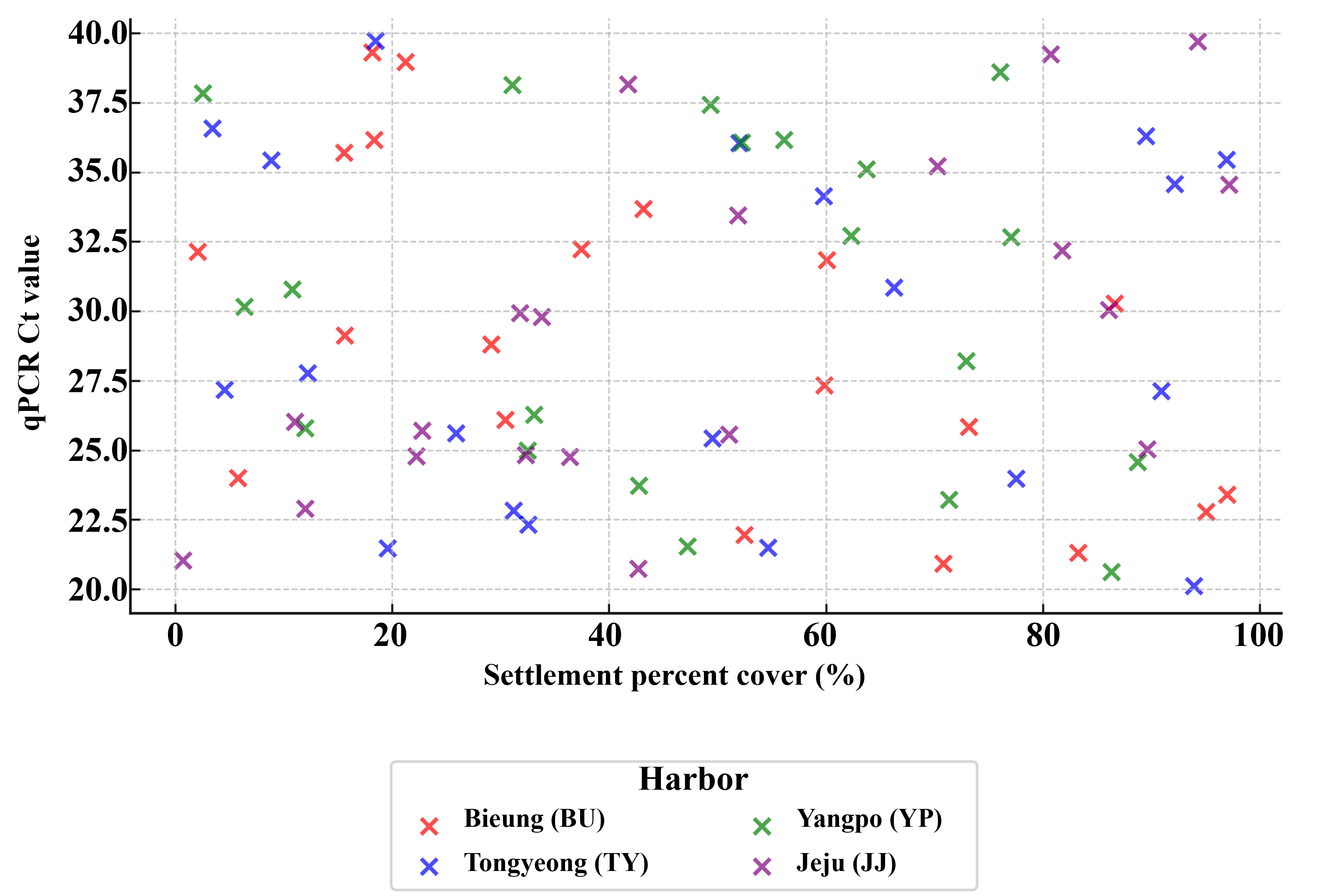
**

**Figure S6. Settlement vs eDNA Ct (2022, matched Harbor×Season**). Scatter plot showing the relationship between settlement percent cover (%) and qPCR cycle threshold (Ct) values across matched harbor × season combinations in 2022. Each color denotes one of the four representative harbors (BU = Bieung, TY = Tongyeong, YP = Yangpo, JJ = Jeju). A negative correlation was observed with lower Ct values corresponding to higher settlement cover.
